# The Association of Military Surgeons

**Published:** 1906-10

**Authors:** 


A Monthly Review of Medicine and Surgery.

*EDITOR:*

WILLIAM WARREN POTTER, M. D.

All communications, whether of a literary or business nature, books for review and exchanges, should be addressed to the editor 284 Franklin St., Buffalo, N. Y.

Vol. LXII. OCTOBER, 1906. No. 3

### The Association of Military Surgeons.

THE fifteenth meeting of the Association of Military Surgeons of the United States was held at Buffalo, September 11-14, and was one of the most successful gatherings in the history of the association. The business meetings were held at the headquarters of the Fourth Brigade N. G. N. Y. in the German Insurance Building and were marked by the excellence of the papers presented and the thoroughness of the discussions which followed. Major James Evelyn Pilcher, secretary of the association and editor of the official journal, in preparing the program brought together a series of papers covering a wide range of topics all of which were of special interest to military surgeons.

The first business was the report of the Enno Sander prize medal board of award. The subject was "The Training of the Medical Officer of the State Forces to best Qualify him for Local Service and for Mobilization with National Troops." The successful essay was written by Major Pilcher, an abstract of which will be published in the Journal at an early day. The essay is one of great value and seems to point a way out of the manifold difficulties which at present appear to stand in the way of highly successful service on the part of the national guard medical officers when called for duty with government troops.

The author believes that harmony with the training of the regular military medical officer is the keynote of the successful adaptation of the medical officer of the state forces to the demands of the service, closely followed by the full recognition of military medicine, surgery and hygiene as a distinct professional specialty, requiring thorough qualification in (I) the special professional knowledge included under (1) military medicine, (2) military surgery, (3) military hygiene; (II) medico-military administration, including (1) the selection of men, (2) definite organization of work, (3) the command of men—(a) the hospital corps, (b) the sick—(4) camp location and organization, (5) field hospital construction and composition, (6) the instruction and drill of the sanitary soldier, (7) "paper work;" (III) the evolution and maintenance of a medico-military esprit de corps, (1) national patriotism and state loyalty, (2) contact with men and literature.

A paper which attracted much attention was that on Tendon tissue versus catgut, read by Dr. Senn, of Chicago. Two papers of superior merit were those by Capt. J. Carlisle De Vries, of Brooklyn, on "Hand and instrument disinfection," and "A sanitary canteen" presented with model of a very serviceable canteen by Dr. Harold D. Corbusier, late U. S. Army.

What undoubtedly was the most important paper of the entire meeting was a preliminary report on uncinariasis in Porto Rico by Capt. Bailey K. Ashford, U. S. Army, read in Capt. Bailey's absence by Capt. P. C. Fauntleroy. The report showed great progress in the investigation of the disease and revealed hitherto unsuspected conditions. The opinions expressed, backed by post mortem evidence and numerous microphotographs of marvellous delicacy in detail, will have a strong bearing on the views to be taken of the condition. When the final report is made it will be the one authority and with the evidence at hand will be conclusive. There will apparently be nothing left for future investigators.

Major L. L. Seaman's paper on "The ration of the soldier" was productive of more discussion than any paper read at the meeting, and the arguments which followed lasted well into an hour, the chief figures in the debate being Dr. Seaman and Col. McPherson of the British army.

The social side of the meeting was brilliantly carried out and it is to the credit of the local committee of which Hon. Herbert P. Bissell was the chairman, that no promise of entertainment was forgot. Dr. Lucien Howe gave a luncheon at his residence at which the Buffalo Academy of Medicine met the officers and foreign delegates and the Buffalo Club gave a brilliant reception to the members of the association. The president, Lieut. Col A. H. Briggs and president-elect Col. Vallery Havard, U. S. A., with representatives of the army, navy and marine hospital service, were entertained at luncheon at the Iroquois by a Buffalo member.

The day following the last business meeting was given up to a trip to Niagara Falls by boat, a gorge ride and luncheon at Niagara Falls.

In the ordinary course of events the presidency for the coming year belonged to the army and would have been filled by Brigadier General Robert M. O'Reilly, surgeon general. Gen. O'Reilly was unable to be present however, and Col. Vallery Havard was made president in his place. Other officers elected were as follows: First vice-president, Rear Admiral Presley M. Rixey, U. S. N.; second vice-president, Assistant Surgeon General George Tully Vaughan, P. H. & M. H. S.; third vice-president, Col. J. K. Weaver, of Pennsylvania National Guard; secretary, Major James Evelyn Pilcher, U. S. V.; assistant secretary, Capt. J. J. Carlisle DeVries, N. G. N. Y.; treasurer, Maj. Herbert A. Arnold, N. G. Pa.

In his retiring speech, Lieut. Col. A. H. Briggs spoke feelingly of the pleasant relations which had existed during the year he acted as president of the association and bid official farewell to his associates in simple, eloquent words. In assuming the presidency Col. Havard predicted great growth for the association and urged upon the members a continuance of the serious work which has marked the proceedings since its inception.

The only standing committee in which a change is announced is the literary committee, which is made up as follows: Maj. Charles F. Mason, U. S. Army; Surgeon William Braisterd, U. S. N.; Assistant surgeon W. C. Rucker, P. H. & M. H. S.; Capt. Rowe, N. G. Ill.; Dr. N. W. Wilson, late, U. S. Army; Capt. J. Carlisle DeVries, N. G. N. Y.

The next meeting will be held at Norfolk, Va., in October, 1907. The program of the meeting follows:

Further remarks on, and care of gunshot wounds of the abdomen, with special reference to the treatment of such wounds of the urinary bladder. By Former Assistant Surgeon General George Tully Vaughan, P. H. & M. H. S.

Tendon Tissue versus Catgut. By Colonel Nicholas Senn, Illinois N. G.

Hand and Instrument Disinfection. By Captain J. Carlisle De Vries, N. G. N. Y.

Clinical Report of the Use of Yeast in Genito-Urinary Work and Gynecology. By Captain Thomas Page Grant, Kentucky S. G.

Some French Impressions of the Surgery of the Russo-Japanese War. By Captain Charles S. Butler, M. V. M.

A Geometrical Cyrtometer for the Easy Application of Chipault's Method of Cranio-Cerebral Localization. By Surgeon William Hemphill Bell, U. S. Navy.

The Brain a Good Field for Surgery, as Shown by its Disregard for Traumatism. By Captain Charles D. Center, Illinois N. G.

Surgical Treatment of Dysentery. By Surgeon Holcomb C. Curl, U. S. Navy.

Wounds of the Colon Treated without Operation. By Major P. R. Egan, U. S. Army.

The Use of Mercurial Bichloride in Surgery. By acting Assistant Surgeon Montafix W. Houghton, P. H. & M. H. S.

The Classification and Treatment of Burns. By Surgeon Charles P. Kindleberger, U. S. Navy.

Firing Tests and Experiences with the Small Caliber Rifle. By Generalarzt Dr. A. Körting. Translated by Captain William Lassiter, U. S. Army.

A Case of Multiple Gunshot Wound. By Lieutenant Leon T. Le Wald, U. S. Army.

Fractures of the Spine. By Surgeon William P. McIntosh, P. H. & M. H. S.

Surgery at the First Aid Station. By Colonel H. Nimier, French Army.

A Case of Fracture of the Skull. By Captain Samuel M. Waterhouse, U. S. Army.

The Results of a Year's surgical Work in the Old Cavite Hospital. By Passed Assistant Surgeon Ulys. R. Webb, U. S. Navy.

Public Meeting, Y. M. C. A. Auditorium. Reception at Lafayette Hotel.

Defective Vision as a Cause of Rejection for Military Service. By Captain Ernest L. Ruffner, U. S. Army.

Febrile Icterus in Port Townsend. By Surgeon W. G. Stimpson, P. H. & M. H. S.

The Influence of Professor Burggraeve upon Modern Therapeutics; with Especial Reference to Military Practice. By Dr. William T. Thackeray, formerly U. S. Army.

Identification by Finger Prints. By Surgeon Charles Poindexter Wertenbaker, P. H. & M. H. S.

Splenic Abscess as a Not Uncommon Complication of Grave Malarial Infection. By Surgeon William Hemphill Bell, U. S. Navy.

Report of an Epidemic of Cerebro-Spinal Meningitis at the U. S. Naval Training Station, Newport, R. I., with a Discussion of the Disease in Relation to Military Establishments. By Surgeon Norman Jerome Blackwood, U. S. N., Surgeon Middleton Semmes Guest, U. S. N., Passed Assistant Surgeon John Hooe Iden, U. S. N., and Passed Assistant Surgeon John Land Neilson, U. S. N.

Infantile Scorbutus Presenting Pseudo-paralysis as the Predominating Symptom. Report of case occurring in the Philippines. By Captain Weston P. Chamberlain, U. S. Army.

A Study of West Indian Bilharziosis. By Passed Assistant Surgeon Richmond Cranston Holcomb, U. S. Navy.

The Opportunities Afforded by the Military Medical Services for Applying Statistical Methods in Therapeutics. By Surgeon (Lieutenant Commander) W. F. Arnold, U. S. Navy, Retired.

Luncheon for the Officers of the Association and the Foreign Delegates at the residence of Dr. C. W. Howe.

The Load of the Foot Soldier. By Colonel Henri Mareschal, French Army.

The Technique of A Yellow Fever Campaign. By Assistant Surgeon William Colby Rucker, P. H. & M. H. S.

Where Treatment of all infected is the Surest Prophylactic Measure—the Problem of Epidemic Uncinariasis in Porto Rico. By Captain Bailey K. Ashford, U. S. Army.

Natural versus Mechanical Ventilation of Hospitals. By Surgeon Preston H. Bailhache, P. H. & M. H. S.

Tuberculosis in the Military Service. By Major George E. Bushnell, U. S. Army.

Notes on Recruiting. By Major Henry I. Raymond, U. S. Army.

The Medical and Sanitary Questions Met in Everyday Life on a Cruising Ship of the Navy. By Medical Director Manly H. Simons, U. S. Navy.

Tropical Hygiene in Reference to Clothing, Houses, Routine and Diet. By Passed Assistant Surgeon Allan Stuart, U. S. Navy.

Report of Two Cases of Apparent Death from Drowning, Showing the Efficiency of Prolonged Artificial Respiration. By Assistant Surgeon John W. Trask, P. H. & M. H. S.

Reception to Officers, Members and Foreign Delegates at the Buffalo Club.

Notes on the Russian Red Cross. By Colonel Vallery Havard, U. S. Army.

The Ration of the Soldier. By Major Louis Livingston Seaman, U. S. V. E.

Recent Observations on Practical Camp Sanitation with Mobilized Troops in Field and Semi-Permanent Camps. By Lieutenant Colonel Louis M. Maus, U. S. Army, and Captain W. J. L. Lyster, U. S. Army.

The work of the Hospital Corps of the Oregon National Guard at the San Francisco Disaster. By Captain William E. Carll, Oregon N. G.

The United States Marine Hospital at San Francisco in the Great Earthquake and Fire of 1906. By Surgeon Henry W. Sawtelle, P. H. & M. H. S.

The Sanitary Department of the Russian Armies in the Far East,—Its Method of Evacuation of the Sick and Wounded. By Colonel John Van Rensselaer Hoff, U. S. Army.

The Military Medical Department in the South West African Uprising of 1904-1905. By Stabsarzt Dr. Kuhn, German Army. Translated by Lieutenant George J. Ogden, U. S. Army.

Experiences with the New York Volunteer Cavalry during the Spanish-American War. By Captain Medwin Leale, N. G. N. Y.

The Work of the Medical Department at Camp Roosevelt. By the Medical Officers on Duty at the Camp of Instruction.

The Workings of the Ancon Hospital, Isthmus of Panama. By Major John L. Phillips, U. S. Army.

The Bravery of the Medical Officer. By Major James Evelyn Pilcher, U. S. V., Captain U. S. Army.

A First Aid Splint Packet. By Surgeon Raymond Spear, U. S. Navy.

Note on the Military Medical Service of the Teutonic Order. By Stabsarzt Dr. Johann Steiner, Austro-Hungarian Service.

The Medical Officer and the Line—A Reminiscence. By Major R. S. Woodson, U. S. Army.

A Sanitary Canteen. By Harold D. Corbusier, late U. S. Army.

Military Medical Chest for use on Mountainous Roads. By Lieutenant Colonel Alejandro Ross, Mexican Army.

A Case for the Chemical Analysis of Water in the Field. By Major Edgar F. Sommer, Indiana N. G.

Equipment of a National Guard Field Hospital. By Lieutenant Colonel Eugene A. Smith, N. G. N. Y.

A Consideration of Recent Views on the Work of the Medical Department in Naval Warfare. By Medical Director John Cropper Wise, U. S. Navy.

Laboratory Work Aboard Ship. By Passed Assistant Surgeon Alfred William Balch, U. S. Navy.

Proposal for a National Medical Service or Department of Public Health. By Surgeon Sheldon Guthrie Evans, U. S. Navy.

A New Field Latrine which fulfills the Requirements for Troops in Active Service where such Device is Necessary. By Captain Frederick C. Herrick, Ohio N. G.

A Description of an Electric Incubator for use aboard Ship. By Passed Assistant Surgeon Richmond C. Holcomb, U. S. Navy.

Steamship and Trolley Excursion down the Niagara River and along its banks.

Luncheon at Niagara Falls tendered by Major M. B. Butler.

